# New Insights Into the Lineage-Specific Expansion and Functional Diversification of Lamprey AID/APOBEC Family

**DOI:** 10.3389/fimmu.2022.822616

**Published:** 2022-03-11

**Authors:** Yan Chen, Lingjie Luo, Lisi Deng, Xiaoxue Tian, Shangwu Chen, Anlong Xu, Shaochun Yuan

**Affiliations:** ^1^ Guangdong Key Laboratory of Pharmaceutical Functional Genes, Southern Laboratory of Ocean Science and Engineering (Zhuhai), State Key Laboratory of Biocontrol, School of Life Sciences, Sun Yat-sen University, Guangzhou, China; ^2^ Laboratory for Marine Biology and Biotechnology, Qingdao National Laboratory for Marine Science and Technology, Qingdao, China; ^3^ School of Life Sciences, Beijing University of Chinese Medicine, Beijing, China

**Keywords:** AID/APOBEC, variable lymphocyte receptor, lamprey, DNA deamination, expansion, virus evolution

## Abstract

The AID/APOBEC family which converts cytidine to uridine on RNA or DNA experienced dynamic expansion in primates in order to resist exogenous viruses and endogenous retrotransposons. Recently, expansion of AID/APOBEC-like homologs has also been observed in the extant jawless vertebrate lamprey. To reveal what causes such expansion and leads to the functional diversification of lamprey cytosine deaminases (CDAs), we reassessed the *CDA* genes in *Lethenteron japonicum* (Lj). We first confirmed the expansion of *LjCDA1L1* (*CDA1-like 1*) genes and found the expression correlation of LjCDA2 and LjCDA1L2 with LjVLRs (variable lymphocyte receptors). Among up to 14 LjCDA1L1 proteins, LjCDA1L1_4a has an extremely high deamination activity on ssDNA and buDNA and, unexpectedly, on dsDNA. LjCDA1L1s can also restrict the infection of HSV-1 particles. Thus, the arms race between the host and pathogens along with the recruitment by VLR assembly may participate together to form a driving force in the expansion and diversification of the lamprey AID/APOBEC family.

## Introduction

The AID (activation-induced deaminase)/APOBEC (apolipoprotein B mRNA editing enzyme, catalytic polypeptide-like) family, including AID, APOBEC1, APOBEC2, APOBEC3s, and APOBEC4, is a class of deaminase proteins that can catalyze C-to-U deamination on single-stranded DNA (ssDNA) or RNA, leading to point mutations in genes or generation of double-strand breaks (DSB) on DNA (1). All AID/APOBEC members contain a conserved deaminase domain with an active center composed of **H**x_1_ **E**x_23–28_ **C**x_2–4_ **C** (x stands for any amino acid) motif and play important roles in many distinct biological processes (2). For instance, APOBEC1 was shown to deaminate C6666 on apolipoprotein B (apoB) pre-mRNA into uracil, resulting in the production of two apolipoproteins with different lengths (apob48 and apob100) for transporting different lipids *in vivo* (3). APOBEC1 has been shown to modify the 3′-UTR regions *via* cytidine deamination in the transcripts of many genes (4). Recently, it has also been found that mutation of the 5′-TpC-3′ site in a variety of cancer cells may be caused by AID/APOBEC proteins (5).

Besides their involvement in lipid metabolism, AID/APOBEC proteins, particularly the AID and APOBEC3 subfamily, were strongly indicated to play important roles in innate and adaptive immune responses (6, 7). Human APOBEC3s, mainly expressed in the peripheral blood and immune center, play a wide range of functions in restricting the activities of exogenous viruses and endogenous retrotransposons (8, 9). For example, APOBEC3G participates in anti-HIV-1 infection by mutating cytidine on HIV-1 reversely transcribed DNA before HIV nucleic acid is integrated into the host cell genome (10). APOBEC3A and APOBEC3B restrict DNA viruses that replicate in the nucleus, such as hepatitis B virus (HBV) and human papillomavirus (HPV) (11, 12). In addition to their antiviral functions, APOBEC3s have the functions of inhibiting retrotransposons, such as Ty1 LTR retrotransposons, Alu and MusD elements, and LINE-1 retrotransposons (13). Due to their important roles in antiviral defense, the amplification of APOBEC3 from one to a subfamily with seven members A3A–A3H in primates is considered a result of co-evolution between the host and the retroviruses (14, 15).

Another immunologic process in which AID/APOBEC proteins are involved is the diversification and maturation of antibodies (2). In jawed vertebrates, B lymphocytes expressed AID which can mediate Ig class switching and somatic hypermutation to increase the specificity and affinity of antibodies (16, 17). AID can also mediate the gene conversion by deamination of cytosine on ssDNA of Ig locus in chickens, dogs, and rabbits (16, 17). AID-induced class switching and somatic hypermutation are mainly carried out by the following steps. The variable region of Ig recruits AID and co-factor replication protein A (RPA) during transcription. RPA stabilizes ssDNA, while AID mediates C-U transitions (18). Subsequently, under the action of uracil-DNA glycosylase (UNG), uracil is removed and the nucleotide-free site can be repaired by any A/T/G/C base (19, 20). If the UNG protein is absent, uracil pairs with A, making the C:G pairing to become A:T pairing, leading to the diversification and maturation of Ig (19).

In jawless vertebrates, two AID/APOBEC-like molecules, PmCDA1 and PmCDA2, from lamprey *Petromyzon marinus* have been shown to express in VLRA/C and VLRB cells, respectively, leading to the hypothesis that PmCDA1 and PmCDA2 may mediate the assembly of VLR genes in jawless vertebrates (21, 22). In 2018, a joint work from Boehm and Larijani reported the striking expansion, diversification, and individual copy number variations (CNVs) of the lamprey AID/APOBEC family (23). Further functional investigations showed that VLRB but not VLRA and VLRC assembly fails in CDA2 lacking lamprey larvae, providing direct evidence that CDA2 is essential for the elaboration of VLRB gene repertoires in jawless vertebrates (24). Outside vertebrates, a family of genes with similarities in amino acid sequences and enzymatic activities to the vertebrate AID/APOBEC family has also been identified in two divergent invertebrate phyla, the echinoderm *Strongylocentrotus purpuratus* and the brachiopod *Lingula anatine* by Sebastian D. Fugmann’s group (25). These ancient enzymes are enriched in tissues undergoing constant and direct interactions with microbes, suggesting that AID/APOBEC proteins and their function in immunity emerged far earlier than previously thought (25).

In order to shed new insights into the co-evolution of the AID/APOBEC family with the immune system and to understand well the expansion and functional diversification of lamprey AID/APOBEC family, we restudied this family by focusing on Japanese lamprey *L. japonicum.* We suggested that the activity of bilateral retrotransposons that resided on the flanking genomic sequences of *LjCDA1* and *LjCDA1L1* genes may be responsible for their CNVs and genetic exclusion. We further reported that a cytidine deaminase named as LjCDA1L_4a has extremely high deaminase activity not only on ssDNA and bubble DNA (buDNA) but also on double-stranded DNA (dsDNA), providing potential advancement of single base editing technology. Finally, we revealed that LjCDA1L1s play critical roles in antiviral response, shedding new light on the co-evolution of the AID/APOBEC family with pathogens.

## Materials and Methods

### Animals, Cells, Strains, and Virus

Adult Japanese lampreys (*L. japonicum*), 25–30 cm long, were obtained from Harbin, China. The harvested lampreys were maintained at 16°C in a lab. *Pelteobagrus fulvidraco* were used to feed lamprey adults daily. Blood was drained from tail-severed adult lamprey and collected into anticoagulant tubes. Buffy coat leukocytes were collected with a peripheral blood lymphocyte separation kit (Solarbio, Beijing, China). Granulocytes, monocytes, and lymphocyte-like cells were sorted into 15 ml tubes by fluorescence-activated cell sorting (FACS).

*Escherichia coli* strain BL21 was purchased from TaKaRa. Herpes simplex virus-1 (HSV-1) was preserved in our lab. HEK293T cells and Vero cells were cultured in DMEM (Gibco, USA) with 10% (vol/vol) fetal bovine serum (Gibco, USA) at 37°C, 5% CO_2_. Transient transfection was conducted with jetPRIME (Polyplus Transfection, France) according to the manufacturer’s instructions.

### Southern Blotting

Genomic DNA of lamprey liver was firstly extracted by phenol chloroform, then digested with *Bam*HI or *Hin*dIII at 37°C, overnight. The digested genomic DNA was then separated in 0.8% agarose gel. The gels were blotted to a positively charged nylon membrane by capillary transfer with 20× SSC. The DNA was fixed to the nylon membrane by UV crosslinking, prehybridization, and hybridization according to the manufacturer’s instruction of DIG High Prime DNA Labeling and Detection Starter Kit II (Roche 11585614910, Switzerland). Bands were recorded on X-ray films (Kodak, Xiamen, China).

### Semiquantitative Reverse Transcription PCR

The cloned LjAID/APOBEC cDNA sequences were deposited in the GenBank database under accession numbers from MT240947 to MT240962 and from OM218945 to OM218956. Granulocytes, lymphocyte-like cells, and monocytes were selected by FACS. Total RNAs of distinct lamprey tissues and cells were extracted using TRIzol reagent (TaKaRa, Japan) and first-strand cDNAs were synthesized with oligo(dT) primers (TaKaRa, Japan) according to the manufacturer’s instructions. Then the distribution of lamprey *LjAID/APOBEC* and *VLR* genes was detected by semiquantitative reverse transcription PCR (SqRT-PCR). Primers are described in **Supplementary Information Table S1**. All samples were anonymously coded in accordance with local ethical guidelines (as stipulated by the Declaration of Helsinki), and the protocol was approved by the Review Board of School of Life Sciences, Sun Yat-sen University.

### Plasmid Construction

HsAID (NM_020661.4), HsAPOBEC3G (NM_021822.4), and HsAPOBEC3A (NM_145699.4) were synthesized and cloned into expression vector pcDNA3.1 from Convenience Biology Corporation (Changzhou, China). For the expression assays in 293T cells, the open reading frames of LjAID/APOBEC were inserted into the expression vectors pEZ-M12 and pEZ-M06 (Genecopoeia, Guangzhou, China). For confocal immunofluorescence microscopy, the coding sequences of LjCDA2 and LjCDA1L2 spliceosomes were inserted into the expression vector pEGFP-N1 (Clontech, USA). For the prokaryotic expression, the coding sequences of LjAID/APOBEC, HsAID, and HsA3G were inserted into the His-tagged pET-28a(+) expression vector. For protein purification, the coding sequences of LjCDA1L1s and HsA3G were cloned into pTT5 with an N-terminal maltose-binding protein (MBP) tag.

### Subcellular Location of LjAID/APOBEC

After transfection by LjAID/APOBEC expression plasmids for 24 h, the 293T cells were harvested. Then, the nuclear and cytoplasmic portions were extracted according to the manufacturer’s instruction of NE-PER™ Nuclear and Cytoplasmic Extraction Reagents (Thermo: #78833, USA) and the concentrations of protein were measured by BCA kit (Thermo, USA). A total of 20 μg of protein mixture per sample was separated on 12% SDS–PAGE gel. Proteins were transferred to PVDF membranes (Bio-Rad, USA) and further incubated with the appropriate antibodies. Bands were revealed with Immobilon ECL kit (Millipore, USA) and recorded on X-ray films (Kodak, Xiamen, China).

### Confocal Immunofluorescence Microscopy

After transfection by the expression plasmids of EGFP-tagged HsA3G, alternative splicing variants of CDA2 and CDA1L2 for 24 h, the 293T cells were fixed in 4% paraformaldehyde, then washed and stained for 5 min with DAPI (Sigma, USA). Imaging of the cells was carried out using Leica TCS SP8 confocal laser microscopy under a ×100 oil objective.

### CRISPR/cas9-Mediated Gene Editing in *Escherichia coli* Strain BL21

gRNA sequences (#1: 5′-TGAAGGGTATTAAGAAAATAAGG-3′ and #2: 5′-CTCGCTGGCGACGGTCTGAAGGG-3′) targeting the *ung* gene of BL21 were predicted *via* http://crispr.mit.edu/http://www.rgenome.net/cas-designer. CRISPR/cas9-mediated *ung* gene editing in BL21 was conducted with CRISPR/cas9-mediated gene editing kit (Inovogen, China) according to the manufacturer’s instructions (Cat. No. CR3010-S).

### Bacterial Rifampicin Resistance Assays

Prokaryotic expression constructs of LjAID/APOBEC, HsAID, and HsA3G were firstly transformed into BL21 and BL21*^ung−/−^* cells. Then, HsAID- and HsA3G-expressing cells were grown overnight at 37°C, while LjAID/APOBEC-expressing cells were incubated at 16°C in LB medium supplemented with 20 μg/ml kanamycin and 0.1 mM isopropylthiogalactoside (IPTG). After an overnight shaking, the bacterial solutions were applied to the rifampicin (100 μg/ml)-containing plates in IPTG induction. Mutation frequencies were measured by determining the median number of colony-forming units that survived per 10^9^ viable cells plated. A total of 50–70 *rpoB* mutants per sample were then selected to further determine the mutation sites of the *rpoB* genes by PCR using the primer pair of 5′-TTGGCGAAATGGCGGAAAACC-3′ and 5′-CACCGACGGATACCACCTGCG-3′. Then, the PCR products were subjected to sequencing.

### *In-Vitro* Cytidine Deamination Assay

Sixteen short ssDNA substrates labeled with CY5 were synthesized by Invitrogen. Eight buDNA substrates were generated by annealing with a threefold excess of the partly complementary strand, while dsDNA substrates were generated according to annealing with threefold excess of the completely complementary strand.

After transfection with 2 μg plasmid for 24 h, the 293T cells in six-well plates were harvested. Soluble whole-cell extracts (WCE) were prepared using the native cell lysate (Invent, USA). The clarified lysate was used for DNA deaminase activity assays. A total volume of 10 μl, containing 5 μl WCE and 0.16 μM CY5-labeled substrates in phosphate buffer (20 mM Tris–HCl, 1 mM EDTA, 1 mM DTT, pH 7.5), was incubated together for 3 h at 37°C and treated with 100 mM NaOH for 10 min at 95°C. Then, a 10-μl reaction buffer was separated directly in 20% denaturing urea-PAGE. The gels were visualized using a CY5 Imager on Quantity One software (Bio-Rad, USA). HsAID and HsA3G were used as positive controls.

For temperature-dependent reactions, a total volume of 10 μl, containing 5 μl WCE and 0.16 μM CY5-labeled substrates in phosphate buffer (20 mM Tris–HCl, 1 mM EDTA, 1 mM DTT, pH 7.5), was incubated together at 14°C, 22°C, and 37°C for 3 or 12 h, respectively. For pH-dependent reactions, the substrates were incubated with WCE in pH 6.0–8.0 at 37°C for 3 h. After inactivating at 85°C for 20 min, the solution was incubated with 2 units of uracil DNA glycosylase (UDG, New England Biolabs, USA) at 37°C for 30 min and treated with 100 mM NaOH for 10 min at 95°C.

### Protein Purification and Deamination Assay on dsDNA

The open reading frames (ORFs) of LjCDA1L1_4a, LjCDA1L1_2a, LjCDA1L1_1a, and HsA3G were cloned into pTT5 with an MBP tag and transfected separately into 293T cells using PEI (Sigma, USA). Cells were harvested at 48 h after transfection, frozen at −80°C, and then resuspended in lysis buffer (25 mM Tris, pH 7.5, 1 M KCl, 1 mM EDTA, 1 mM DTT). The protocol for protein purification was described previously (26). In brief, resuspended cells were centrifuged at 12,000*g* for 10 min and the cleared lysate was mixed with pre-equilibrated amylose resin (NEB, USA) for 1 h at 4°C with shaking. Then, the resin was washed with 50 ml lysis buffer and the proteins were eluted with elution buffer (25 mM Tris, pH 7.5, 0.5 M KCl, 1 mM DTT, 40 mM maltose). The eluate was concentrated and dialyzed in dialysis buffer (25 mM Tris, pH 7.5, 150 mM KCl, 2 mM DTT, 10% glycerol) at 4°C. Samples were stored at −80°C. Protein concentration was determined by SDS-PAGE followed by Coomassie blue staining. The purified protein (200 ng in activity buffer) was incubated at 37°C for 3 h with 0.16 μM CY5-labeled dsDNA, UDG (5 U, New England Biolabs, USA), and apurinic/apyrimidinic endonuclease (APE) (5 U, New England Biolabs, USA). Then, 10 μl reaction solution was treated with 100 mM NaOH for 10 min at 95°C and separated directly in 20% denaturing urea-PAGE. HsA3G was used as control.

### Structure Prediction and Phylogenetic Analysis of LjAID/APOBEC

Structure models of LjCDA1L1s were produced by submitting the sequences to the Swiss protein data bank (https://swissmodel.expasy.org/) and Phyre2 (www.sbg.bio.ic.ac.uk/phyre2) using human A3C X-ray (PDB 3vow), A3B X-ray (PDB 5tkm), A3A NMR (PDB 2m65), and A3F-CTD X-ray (PDB 4iou) as templates. The templates were chosen as the best fit by the two databases. All predicted structures of LjCDA1L1s were visualized using PyMOL v1.3 program (http://www.pymol.org/). The neighbor-joining tree of lamprey AID/APOBEC members was constructed with MEGA version 5.05 using the amino acid sequences obtained from NCBI. The numbers at the nodes indicated bootstrap values.

### Experimental Passages for HSV-1

Flag-tagged vectors were overexpressed in 293T cells for 24 h. Then, the cells were infected by HSV-1 (MOI = 0.1) and incubated at 37°C, 5% CO_2_ for 48 h. The supernatants were collected and clarified by centrifugation at 2,000*g* prior to titration and purification. This process was repeated twice at MOI 0.1 PFU/sample. The HSV-1 genome was extracted from the first and third passage cells. Then, a total of 461-bp US12 coding sequence was separated and inserted into the pGEM-T vector (Promega, USA) and a total of 22 clones per sample were chosen for Sanger sequencing. Primers are described in **Supplementary Information Table S1**.

Plaque assay was performed by inoculating confluent monolayers of Vero cells with a serially diluted virus allowing the virus to adsorb for 60 min. Following adsorption, an agarose overlay was added and the cells were incubated for 30 h at 37°C, 5% CO_2_. Plaques were visualized by plaque staining using 0.03% Neutral Red Solution (Sigma, USA).

## Results

### Expansion and CNVs of *CDA1L1* Genes in Lamprey *Lethenteron japonicum*

**Figure 1:**
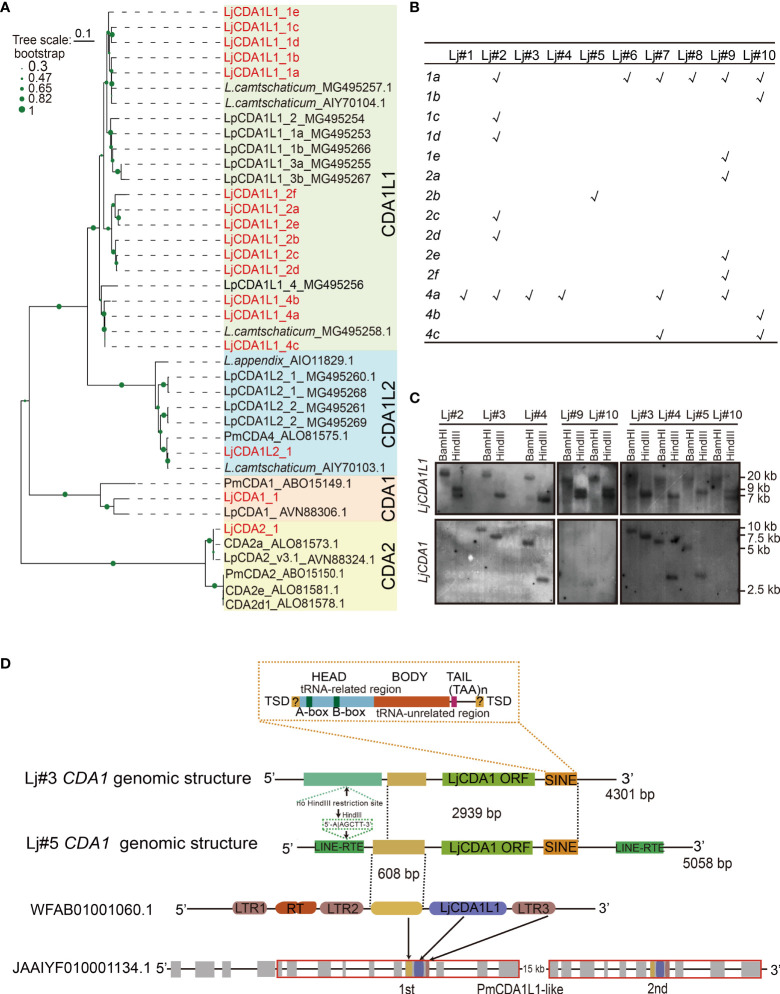
Expansion and genetic exclusion of *LjCDA1* and *LjCDA1L1*. **(A)** Neighbor-joining tree of lamprey AID/APOBEC deaminases. The amino acid sequences of the core region of the cytidine deaminases (CDAs) were aligned using CLUSTALW. Alignments were manually adjusted based on secondary structure predictions. A neighbor-joining tree was calculated using MEGA version 5.05 with 1,000 bootstrap replications. Colored boxes represent distinct lamprey AID/APOBEC deaminases. Species abbreviation: Lj, *Lethenteron japonicum*; Pm, *Petromyzon marinus*; Lp, *Lampetra planeri.* More details are shown in **Figure S1**. **(B)** Distributions of *LjCDA1L1s* in distinct lamprey individuals tested by PCR. Lj#1–Lj#10 indicate 10 distinct individuals. *1a*–*4c* indicate 14 distinct *LjCDA1L1* genes. **(C)** Southern blot analyses of *LjCDA1L1* and *LjCDA1* genes. Genomic DNAs were digested by indicated enzymes before transferring to the Hybond N^+^ nylon membrane. Lj#2–Lj#5, Lj#9, and Lj#10 indicate the individual numbers of Japanese lamprey. **(D)** Genomic organization of lamprey *LjCDA1* gene. SINE, short interspersed nuclear elements. WFAB01001060.1: fragment ID of lamprey *L. camtschaticum*; JAAIYF010001134.1: fragment ID of lamprey *Petromyzon marinus*. RT, reverse transcriptase; LTR1–LTR3, element of LTR retrotransposons.

Gene expansion and CNVs of *CDA1* and *CDA1-like 1* (*CDA1L1*) genes have been reported in lamprey species (23). To further reveal the lineage expansion and the co-evolution of the AID/APOBEC family with the immune system, we reassessed this family in Japanese lamprey *L. japonicum* (*Lj*). We isolated 14 CDA1L1 sequences from 10 distinct adults of *L. japonicum*. These sequences can be clustered with *LpCDA1L1s* identified from *Lampetra planeri*, thus named as *LjCDA1L1_1a-e*, *LjCDA1L1_2a-f*, and *LjCDA1L1_4a*-*c* (**Figure 1A** **;** **Figure S1**). All the sequences are predicted to encode proteins that contain an APOBEC-Z1 domain with a motif composed of X_6-11_ **H**_X_ **E**X_5_XXX_17_ **SWSPC**X_2-4_ **C**X_6_FX_8_LX_5_ **RIY**X_8-11_LX_2_LX_8_XMX_3-4_ (**Figure S2**). Such motif also exists in the vertebrate APOBEC3 subgroup in their catalytic center (14). By performing PCR using genomic DNA from gill tissues of 10 *L. japonicum* individuals, we found CNVs of *LjCDA1L1* among individuals and indicated that *LjCDA1L1_4a* and *LjCDA1L1_1a* are the most common existing *CDA1L1* genes in Japanese lamprey (**Figure 1B**).

To test how such diversity was produced, genomic DNAs from six *L. japonicum* individuals Lj#2~Lj#5, Lj#9, and Lj#10 were subjected to Southern blot hybridization using a probe generated from the sequence of *LjCDA1L1_4a*. As shown by the results, two fragments of ∼7 and ∼9 kb were detected in the *Hin*dIII digested genomes from individuals Lj#2, Lj#9, and Lj#10, while only one fragment with different sizes can be detected in *Hin*dIII digested genomes from individuals Lj#3, Lj#4, and Lj#5 (**Figure 1C**). Since LjCDA1L1 proteins with high sequence identity are all encoded by a single exon and their coding sequences do not contain *Hin*dIII restriction sites, different bands detected from genomes digested by the same restriction enzyme suggested individual CNVs of *LjCDA1L1* genes. Such observation was consistent with the PCR analysis (**Figure 1B**). Like *L. planeri*, only one fragment of ∼20 kb can be identified in genomic DNA digested with *Bam*HI from all six lamprey individuals, suggesting that distinct *LjCDA1L1* copies are arrayed on the same chromosome in *L. japonicum* (**Figure 1C**).

### Genetic Exclusion Between *CDA1* and *CDA1L1s* in Lamprey *Lethenteron japonicum*

Besides LjCDA1L1, we also obtained three CDA1 sequences with 97% identity from six lamprey individuals. Then, genomes from Lj#2~Lj#5, Lj#9, and Lj#10 were also subjected to Southern blot hybridization using the probe generated from the *LjCDA1_1* gene. To our surprise, the hybridized signal can only be detected in individuals Lj#3, Lj#4, and Lj#5, but not in individuals Lj#2, Lj#9, and Lj#10. Besides that, the sizes of the detected fragments were completely different among individuals Lj#3, Lj#4, and Lj#5 (**Figure 1C**). These results indicated that *LjCDA1* not only has CNVs but also may have dynamic locations in the genome of distinct *L. japonicum* individuals. Interestingly, individuals which gained two hybridized bands after *Hin*dIII digestion using *LjCDA1L1_4a* as probe do not have any *LjCDA1* gene in their genome, suggesting the genetic exclusion between *LjCDA1* and some of the *LjCDA1L1* copies in *L. japonicum* (**Figure 1C**). Moreover, the disappeared hybridized band after *Hin*dIII digestion using *LjCDA1L1_4a* as probe was different between individuals Lj#4 and Lj#5, suggesting that the persisted *LjCDA1L1* copies may also be different in these two individuals (**Figure 1C**).

To further reveal how CNVs of *LjCDA1* were produced, we separated the 5′ and 3′ flanking genomic sequences of *LjCDA1* by inverse PCR from individuals Lj#3 and Lj#5. First, we found that both the 5′ and 3′ flanking sequences of *LjCDA1* are different in Lj#3 and Lj#5, demonstrating that *LjCDA1* has changed genomic location among individuals. Due to the absence of a *Hin*dIII restricted site in the 5′ flanking sequence of *LjCDA1* in Lj#3, the hybridized band of *Hin*dIII digested genome in Lj#3 is large than that in Lj#5 (**Figure 1C**). Second, two incomplete LINE (long interrupted nuclear elements) sequences were identified in both the 5′ and 3′ flanking sequences of *LjCDA1* in Lj#5, but not in Lj#3. Downstream of the *LjCDA1* coding sequence, a characteristic SINE (short intersected nuclear element) retrotransposon was identified both in the genomes of Lj#3 and Lj#5. The 5′ head of this SINE retrotransposon contains a typical A-box sequence (TGGCTATGAGAT), 30–35 bp interval sequence, typical B-box sequence (GGTTCGANNCC), and the (TAA)n tail sequence (**Figure 1D**). These observations implied that the activity of the resided retrotransposon may be responsible for the CNVs and dynamic genome insertions of *LjCDA1* among individuals. Third, by the Blastn program, we identified fragment WFAB01001060.1 that contains a *LjCDA1L1* gene flanked by partial LTR (long terminal repeat) sequences from lamprey *L. camtschaticum*. This fragment has a sequence of 608 bp with high identity (92%) to the 5′ flanking sequence of *LjCDA1* (**Figure 1D**). This 608-bp fragment along with the *LjCDA1L1* coding sequence and the downstream LTR3 segment is duplicated in scaffold JAAIYF0100001134.5 from another lamprey species *Petromyzon marinus* (**Figure 1D**). Thus, recent gene duplication of *CDA1L1* is commonly observed in distinct lamprey species.

### Functional Implications of Distinct *AID/APOBEC* Genes in Lamprey

**Figure 2:**
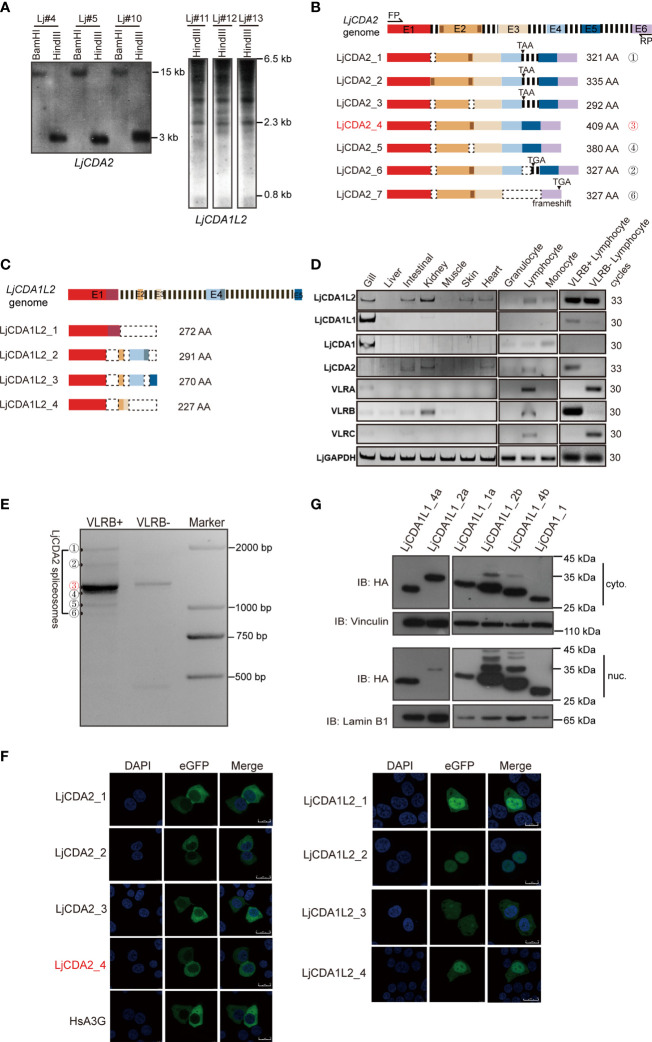
Functional implications of distinct *AID/APOBEC* genes in lamprey. **(A)** Southern blot analyses of *LjCDA2* and *LjCDA1L2* genes. Genomic DNAs were digested by indicated enzymes before transferring to the Hybond N^+^ nylon membrane. Lj#4, Lj#5, and Lj#10–Lj#13 indicate the individual numbers of Japanese lamprey. **(B)** Genomic and mRNA spliceosome structures of the lamprey *LjCDA2* gene. Colored boxes represent exons (E1–E6); dashed lines represent introns; dotted boxes correspond to the absent sequences. FP and RP indicate forward and reverse primer. More details are shown in **Figure S3A**. **(C)** Genomic and mRNA spliceosome structures of the lamprey *LjCDA1L2* gene. Colored boxes represent exons (E1–E5); dashed lines represent introns; dotted boxes correspond to the absent sequences. More details are shown in **Figure S3B**. **(D)** SqRT-PCR to detect the tissue and cell distribution of the lamprey *LjAID/APOBEC* and *LjVLR* genes. **(E)** PCR to detect the alternative splicing variants of LjCDA2 in VLRB-positive and VLRB-negative cells sorted by flow cytometry. ①–⑥ indicate the splicing forms of LjCDA2. **(F)** Subcellular localization of HsA3G, LjCDA2, and LjCDA1L2 splicing forms in 293T cells detected by confocal microscopy. GFP-tagged HsA3G was used as control. **(G)** Western blotting to detect the subcellular localization of LjCDA1_1 and LjCDA1L1 proteins in 293T cells. Results of subcellular locations were representative of three independent biological replicates.

In addition to LjCDA1 and LjCDA1L1, the sequences clustered with LpCDA1L2 and PmCDA2 were also isolated from *L. japonicum* (**Figure 1A** **;** **Figure S1**). Unlike LjCDA1 and LjCDA1L1s which are encoded by a single exon and have CNVs, *LjCDA2* is a single copy, while *LjCDA1L2* was detected to have at least two copies. Although both *LjCDA2* and *LjCDA1L*2 do not have CNVs among individuals (**Figure 2A**), we identified at least seven *LjCDA2* and four *LjCDA1L2* splicing forms from Japanese lamprey tissues (**Figures 2B, C** **;** **Figures S3A, B**). Thus, expansion, genetic exclusion, and alternative splicing of lamprey AID/APOBEC members broadly increase their complexity.

To further reveal the functional implications of the lamprey AID/APOBEC family, we performed SqRT-PCR to detect distinct LjAID/APOBEC members for tissue distributions using cDNAs purified from individual Lj#5. Results showed dominant expression of LjCDA1L1 and LjCDA1 in gills (**Figure 2D**). LjCDA1L1 but not LjCDA1 can also be detected in VLRB-positive cells (**Figure 2D**). LjCDA1L2 was found to be abundant in both VLRB-positive and VLRB-negative lymphocyte-like cells and broadly distributed in the gill, intestine, kidney, skin, and heart (**Figure 2D**). Unlike other CDA homologs, expression of LjCDA2 is highly restricted to VLRB-positive cells (**Figure 2D**). After sequencing the LjCDA2 splicing forms obtained from VLRB-positive cells, we found that LjCDA2_4 (the ③ band) is the main splicing isoform in VLRB cells (**Figures 2B, E** **)**. Given that CDA2 has been demonstrated to participate in the assembly of VLRB, the cytoplasmic location of LjCDA2 splicing forms in 293T cells suggested that they may be cytoplasm and nucleus shuttle proteins like human AID (**Figure 2F**). Subcellular localization of lamprey AID/APOBEC also showed that most of the LjCDA1L1 and LjCDA1 proteins can localize both in the cytosol and nucleus (**Figure 2G**), while LjCDA1L2 splicing forms are predominantly distributed in the nucleus (**Figure 2F**). Thus, the expression profiles and the subcellular locations may suggest the diversified functions of lamprey CDA proteins.

### Cytidine Deaminase Activity of Lamprey AID/APOBEC Proteins in *Escherichia coli* BL21

**Figure 3:**
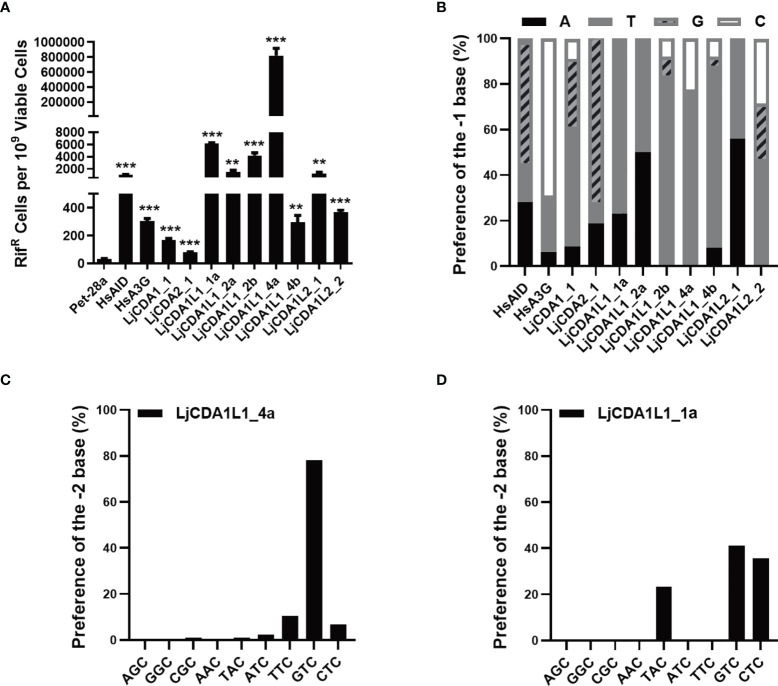
LjAID/APOBEC stimulated *rpoB* mutations in *Escherichia coli.* **(A)** Mutagenic activities of the LjAID/APOBEC proteins in BL21*^ung−/−^ E*. *coli*. Activity was measured by the number of rifampicin-resistant (Rif^R^) colonies per 10^9^ viable cells (number of kanamycin-resistant colonies). Deaminase activities were evaluated against the vector control using an unpaired Student’s *t*-test with Welch’s correction. ***P < 0.0001, **P < 0.001. **(B)** The statistical analysis of the base preference of LjAID/APOBEC family proteins at the −1 position upstream of cytidine deamination site (0). **(C, D)** The preferred trinucleotide of LjCDA1L1_4a and LjCDA1L1_1a on the *rpoB* gene in BL21*^ung−/−^ E. coli*.

To further compare the functional differences among LjAID/APOBEC members, we employed rifampicin resistance (Rif^R^) assay to detect the deaminase activity of LjAID/APOBEC proteins in *E. coli* BL21. Meanwhile, human AID (HsAID) and APOBEC3G (HsA3G) were used as the positive controls, while Pet-28a empty vector was used as the negative control (**Figures S4A, B**). The results of the Rif^R^ assay showed that *E. coli* BL21 cells transformed with the expression vectors containing LjAID/APOBEC coding sequences gained Rif^R^ (**Figure S4C**). BL21 cells expressing LjCDA1L1_4a have the highest mutation frequency on the *rpoB* gene and then followed by the cells expressing LjCDA1L1_1a and LjCDA1L1_2b (**Figure S4C**). BL21 cells expressing LjCDA1L1_2a and LjCDA1L2_1 showed resistance to rifampicin equally. However, BL21 cells expressing LjCDA1L1_4b, LjCDA1_1, and LjCDA2_1 almost could not resist Rif (**Figure S4C**). Since the converted U produced by the AID/APOBEC family can be removed by UNG to make the sites as nucleotide-free sites, which will be replaced by any nucleotides or recognized by nucleotide-free endonuclease, this leads to DNA break. To avoid such influence, we then generated an *ung^−/−^* BL21 strain to repeat the Rif^R^ assays (**Figure S4D**). Results showed that the frequency of Rif^R^ was greatly increased in all LjAID/APOBEC-expressing BL21*^ung−/−^* cells (**Figure 3A**), further indicating that the Rif^R^ is indeed caused by dC→dT or dG→dA shifts on the *rpoB* gene mediated by lamprey AID/APOBEC proteins.

Then, sequencing of the *rpoB* gene from hundreds of independent Rif^R^ colonies confirmed the high frequency of dC→dT and dG→dA transitions in LjAID/APOBEC-expressing BL21*^ung−/−^* cells, but not in cells transformed with empty vector (**Figure S4E**). Analyses of the nucleotides with dC→dT and dG→dA transitions further indicated the dinucleotide preferences of distinct LjAID/APOBEC proteins. As shown in **Figure 3B**, LjCDA1L1s prefer 5′-TC dinucleotide substrate, while LjCDA1L2_1 prefers 5′-AC. LjCDA2_1 prefers 5′-GC dinucleotide substrate like human AID (**Figure 3B**). Analyses of the trinucleotide motifs indicated that the bases of −2 upstream of C (position 0) can also affect the deamination efficiency of lamprey AID/APOBEC proteins. As shown in **Figures 3C, D**, LjCDA1L_4a prefers 5′-GTC, 5′-TTC, and 5′-CTC, while LjCDA1L_1a prefers 5′-GTC, 5′-CTC, and 5′-TAC. Trinucleotide preferences of the other LjAID/APOBEC proteins are shown in **Figure S4F**.

### Cytidine Deaminase Activity of Lamprey AID/APOBEC Proteins on ssDNA and buDNA *In Vitro*

**Figure 4:**
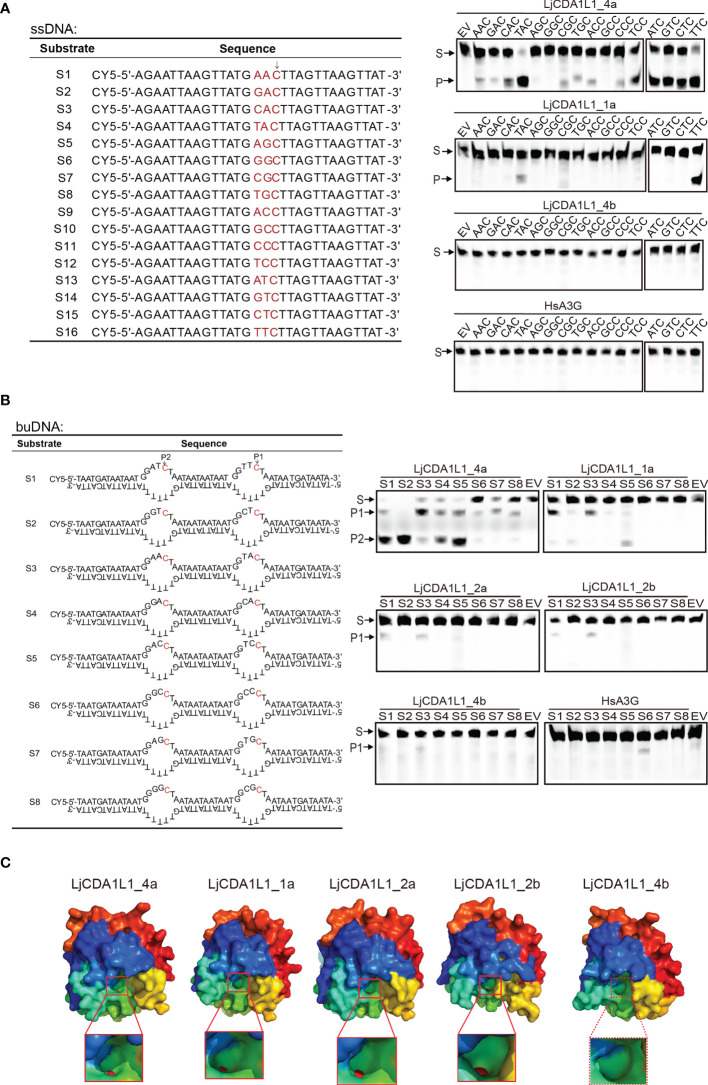
Cytidine deaminase activity of LjAID/APOBEC on single-stranded DNA (ssDNA) and bubble DNA (buDNA) *in vitro*. **(A)** Cytidine deaminase activity of LjAID/APOBEC on ssDNA *in vitro*. The table indicates the used ssDNA substrates labeled with CY5. WCLs of LjAID/APOBEC expressing 293T cells were used to test the enzyme activity. Arrow represents the target C. S, substrate; P, product; EV, empty vector. **(B)** Cytidine deaminase activity of LjAID/APOBEC on buDNA *in vitro*. The table indicates the used buDNA substrates labeled with CY5. WCLs of LjAID/APOBEC expressing 293T cells were used to test the enzyme activity. Arrows represent the target C. S, substrate; P, product; EV, empty vector. **(C)** The predicted crystal structures of LjCDA1L1_4a, LjCDA1L1_1a, LjCDA1L1_2a, LjCDA1L1_2b, and LjCDA1L1_4b using the SWISS-MODEL. Red boxes represent the “pocket.” Red spheres represent zinc ions. All results were representative of three independent biological replicates.

To confirm the enzyme activity and verify the substrate specificity of the lamprey AID/APOBEC family, a total of 16 ssDNAs (30 bp in length) with only one C base were designed as ssDNA substrates for *in-vitro* deamination assays (**Figure 4A**). Expression vectors containing lamprey AID/APOBEC and HsA3G were transfected into 293T cells. Then, cell extracts containing expressed AID/APOBEC proteins were incubated with distinct ssDNA substrates. If lamprey AID/APOBEC proteins can mediate C to U transition, DNA breaks will happen at the transition sites due to the presence of UNG and APE in the 293T cell lysate. Results showed that LjCDA1L1_4a has high deamination activity on trinucleotide motifs of 5′-TAC and 5′-TTC (**Figure 4A**). It also has broad deamination activity on 5′-ATC, 5′-GTC, 5′-CTC, and 5′-TCC. LjCDA1L1_1a also prefers trinucleotide motifs 5′-TTC and 5′-TAC, but almost cannot function on the other tested substrates (**Figure 4A**). Besides LjCDA1L1_4a and LjCDA1L1_1a, almost no deamination activities could be observed for the other tested lamprey AID/APOBEC proteins under the same condition.

Considering that deamination takes place on the Y shape or bubble genomic DNA (buDNA) in the case of transcription, replication, or DNA damage *in vivo*, eight buDNA substrates with distinct trinucleotide motifs were designed to detect the deaminase activity of LjAID/APOBEC proteins (**Figure 4B**). Results showed that HsA3G specifically prefers to deaminate at 5′-CCC with low efficiency in line with the other reports (6). Although LjCDA1L1_4a shows high and broad enzyme activity, it has sequence preferences on 5′-GTC, 5′-ATC, and 5′-ACC on buDNA (**Figure 4B**). Similarly, LjCDA1L1_1a, LjCDA1L1_2a, and LjCDA1L1_2b prefer 5′-TTC or 5′-TAC, suggesting that not only the substrate conformation but also the upstream bases are important for the activity of LjAID/APOBEC family proteins (**Figure 4B**).

To further explain the functional diversification, we then used the SWISS-MODEL to simulate the crystal structures of LjAID/APOBEC proteins. The results showed that LjCDA1L1_4a, LjCDA1L1_1a, LjCDA1L1_2a, and LjCDA1L1_2b can chelate with Zn atom, and the shape of a “pocket” is similar to a dumbbell which allows cytidine to enter (**Figure 4C**). Since the amino acids in the loop structure which stabilizes the active center are important reasons for the activity of AID/APOBEC deaminases (27–30), the inability of LjCDA1L1_4b to chelate with Zn atom may result in its weak deaminase activity as we have tested (**Figure 4C**).

Besides, we investigated the deamination activity of LjCDA1L1s under different pH values and temperatures using the 293T cell extracts containing the overexpressed proteins. Like human A3F-CTD with the optimal pH from 5.5 to 9.5 (31), LjCDA1L1_4a has a wide pH range adaptability (**Figure S5A**). However, LjCDA1L1_1a, LjCDA1L1_2a, and LjCDA1L1_2b prefer approximately neutral and slightly basic pH (6.5–8.0) (**Figure S5A**). The protonation/deprotonation of the side chains of the ionizable residues in loop 1 has been reported to be responsible for the pH-dependent deaminase activities of human AID/APOBEC proteins, such as the residues R25 and E26 in human AID (32). Thus, we speculated that the G32 and R33 residues of LjCDA1L1s may modulate their pH sensitivity. We also investigated the deamination activity of LjCDA1L1s under different temperatures (14°C, 22°C, and 37°C) and actuation duration (3 or 12 h). The results showed that the optimal temperature of LjCDA1L1s is 37°C (**Figure S5B**). However, when the actuation duration was extended to 12 h, the deamination activity of LjCDA1L1s at 22°C is close to that at 37°C (**Figure S5B**). Thus, lamprey CDA deaminases have a wide pH and temperature tolerance.

### Cytidine Deaminase Activity of LjCDA1L1_4a on dsDNA *In Vitro*

**Figure 5:**
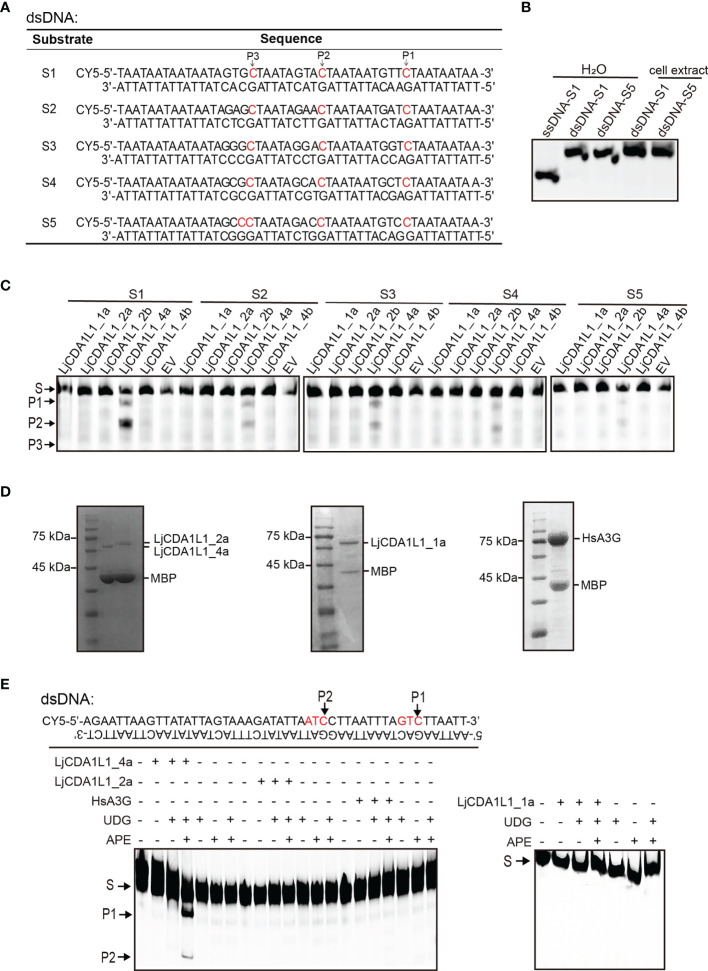
Cytidine deaminase activity of LjCDA1L1s on dsDNA *in vitro*. **(A)** The table indicates the used dsDNA substrates labeled with CY5. Arrows represent the target C. **(B)** The migration of ssDNA and dsDNA detected by native electrophoresis. **(C)** Cytidine deaminase activity of LjCDA1L1s on dsDNA using cell lysates from LjCDA1L1s expressing 293T cells. S, substrate; P, product; EV, empty vector. **(D)** The purified LjCDA1L1_4a, LjCDA1L1_1a, LjCDA1L1_2a, and HsA3G proteins from 293T cells were shown by SDS-PAGE and Coomassie blue staining. **(E)** Cytidine deaminase activity of the purified proteins on dsDNA *in vitro*. Arrows represent the target C. S, substrate; P, product. All results were representative of three independent biological replicates.

When we tested the deamination activity of LjAID/APOBEC on buDNA, substrates with multiple C bases on the buDNA were used in the pre-experiments. Interestingly, fragments with unexpected sizes were generated when substrates were incubated with LjCDA1L1_4a-expressing cell lysates, suggesting that LjCDA1L1_4a may function on the other dC within the dsDNA (**Figure S6A**). To detect if LjCDA1L1_4a indeed has deamination activity on dsDNA, we then constructed three double-stranded (ds) DNA substrates containing multiple dC sites. Using the experimental procedure mentioned above, we found multiple cleaved bands in the catalytic assays (**Figure S6B**). Then, five dsDNA substrates with specific dC sites were designed for further deamination assays (**Figure 5A**). We first used native gel electrophoresis to confirm that these dsDNA oligonucleotides in the 293T cell extract were not broken into ssDNA (**Figure 5B**). Then deamination assays showed that LjCDA1L1_4a but not the other LjAID/APOBEC proteins indeed can function on dsDNA (**Figure 5C**). The deamination of LjCDA1L1_4a on dsDNA also prefers 5′-AC and 5′-TC (**Figure 5C**).

To further eliminate the influence of the other endonucleases in 293T cell lysates, LjCDA1L1_4a, LjCDA1L1_1a, LjCDA1L1_2a, and HsA3G proteins were overexpressed, purified from 293T cells, and then incubated with indicated dsDNA substrates (**Figure 5D**). In the presence of UDG and APE, DNA breaks were observed, further indicating the deamination on dsDNA mediated by LjCDA1L1_4a but not the other tested AID/APOBEC proteins (**Figure 5E**). Thus, LjCDA1L1_4a not only has extremely high deaminase activity on ssDNA and buDNA but also can act on dsDNA. Such activity could largely expand its target sequences and is beneficial for its application on gene editing technology.

### The Antiviral Activity of LjCDA1L1 Subgroup to HSV-1

**Figure 6:**
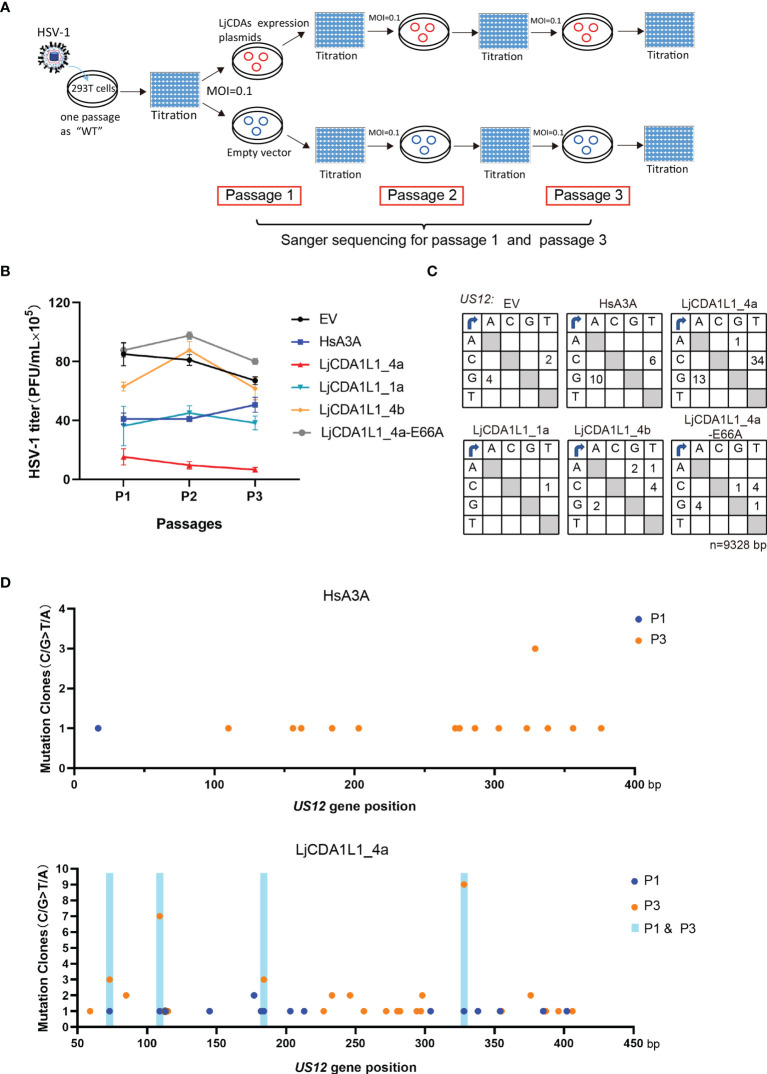
The antiviral activity of the LjCDA1L1 subgroup to HSV-1. **(A)** Schematic diagram of the HSV-1 passaging experiment. 293T cells were transfected with LjCDA expression constructs and then infected by HSV-1 (MOI = 0.1 PFU/cell). Then, HSV-1 particles in the cell supernatants were harvested to infect the fresh 293T cells which were transfected with indicated LjCDA constructs. Such experiment was repeated three times. **(B)** PFUs of HSV-1 harvested from the supernatants of LjAID/APOBEC and HsA3A proteins expressing 293T cells at 48 h after infection by HSV-1 at 0.1 MOI from three passages. Error bars denote ± SD. **(C)** Mutation matrices for the *US12* gene of HSV-1 extracted from 293T cells expressing LjAID/APOBEC and HsA3A proteins from the third passages. *n* = 9,328 bp. **(D)** Mutation clones plotted against nucleotide position of the *US12* gene of HSV-1 extracted from 293T cells expressing LjCDA1L1_4a and HsA3A proteins from passage 1 and passage 3. The mutation sites present only in passage 1 are indicated by blue dots, while the mutation sites present only in passage 3 are indicated by orange dots. The mutation sites present both in passage 1 and passage 3 are indicated by shaded boxes.

HsAPOBEC3s are well-known IFN-stimulated genes (ISGs) which play important roles against viral infection. Given that LjCDA1L1 proteins contain a HsA3s-like zinc-coordination domain which acts on ssDNA and buDNA, we used HSV-1 to explore the antiviral activity of the LjCDA1L1 subgroup. HSV-1 is representative of *Alphaherpesvirinae* that has linear dsDNA genomes and could infect epithelial cells (33). To explore whether LjCDA1L1s can edit the HSV-1 genome in a deaminase-dependent manner, LjAID/APOBEC proteins were overexpressed in 293T cells for 24 h. Then, the cells were infected by HSV-1 [MOI = 0.1 plaque-forming units (PFU)/cell] for further 48 h and the supernatants were collected to assess the replication of the virus on Vero cells. To explore the effects of LjCDA1L1s on the evolution of HSV-1, the harvested HSV-1 particles were then used to infect the fresh 293T cells in which LjAID/APOBEC proteins were overexpressed. Such experimental procedure was repeated three times at MOI = 0.1 PFU/cell (**Figure 6A**). As shown in **Figure 6B**, the titer of the virus released from LjCDA1L1_4a-expressing cells was significantly reduced (by 10-fold) when compared with the control (EV) cells in three passages. For LjCDA1L1_1a and HsA3A, a slight decrease of viral titer in the first and second passages was also observed (**Figure 6B**). By contrast, LjCDA1L1_4b and LjCDA1L1_4a-E66A, which lose the deaminase activity, both had no significant impact on the replication of HSV-1 when compared with controls in all passages (**Figure 6B**). The other deaminases including LjCDA1_1, LjCDA2_4, and LjCDA1L2_1 also had no significant impact on the replication of HSV-1 (**Figures S7A, C**). Given that LjCDA1L1 proteins are quite different from each other at deamination activity, we suggested that the antiviral effect on HSV-1 of LjAID/APOBEC proteins may be deaminase-dependent. Thus, total DNA of HSV-1 was extracted from the first and the third passages as template to clone the immediate-early gene *US1*2 for sequencing. *US12* encodes infected cell polypeptide 47 (ICP47) involved in immune escape (34). As expected, we found that editing in double strands of the HSV-1 *US1*2 gene was obviously in LjCDA1L1_4a-overexpressed cells (**Figure 6C** **;** **Figure S7B**). Sequence comparison between the first passages and the third passages indicated that the mutation frequency of the third passages mediated by LjCDA1L1_4a and HsA3A is higher than that of the first passages (**Figure 6D** **;** **Figure S7D**). Moreover, most mutations found in both passages 1 and 3 were non-synonymous (**Figure S7E**), and some of the non-synonymous mutations mediated by LjCDA1L1_4a were accumulated, suggesting the effects of LjCDA1L1s on the evolution of HSV-1 (**Figure 6D**). Although the substrates of LjCDA1L1s are primarily ssDNA and buDNA, it can also act on dsDNA oligonucleotides with low efficiency. Thus, we expected that nuclear LjCDA1L1s can target the single-stranded HSV-1 DNA during viral DNA synthesis or transcription, as well as viral naked DNA in the nucleus, which may be beneficial to the host in the arms race with the viruses. Similarly, human AID can target dsDNA with multiple topologies in a transcription-independent manner or breathing ssDNA patches occurring in de-chromatinized dsDNA *in vivo* (35, 36). Moreover, the activity of human AID is tightly regulated in activated B cells and restricted to the immunoglobulin (Ig) loci (37). Since high deamination activity on dsDNA in the nucleus may influence the genome stability, the activity of LjCDA1L1_4a on dsDNA should be tightly regulated under long-term adaptive pressure.

## Discussion

In a striking difference to Ig-mediated adaptive immune systems in jawed vertebrates, the diversity of variable lymphocyte receptors (VLR) was revealed to have an alternative adaptive immune system in jawless vertebrate lamprey (38). The VLR repertoire up to 10^14^ was thought to be produced by a rearrangement process like gene conversion (21). After the discovery of VLR, two APOBEC/AID homologs, PmCDA1 and PmCDA2, were identified in lamprey *P. marinus* and implicated to mediate the rearrangement of VLRs (21). Recently, two additional subgroups, *CDA1L1* and *CDA1L2* evolved from *CDA1*, were identified in another lamprey species *L. planeri* (23). Moreover, *LpCDA1* and *LpCDA1L1_4* were found to be genetically exclusive in *L. planeri*. Here, we further confirmed the genetic exclusion between *CDA1* and *CDA1L1* in another lamprey species, the *L. japonicum*. Moreover, the genetic exclusion is not only existing between *LjCDA1* and *LjCDA1L1_4*, but also the other *LjCDA1L1* copies. Analyses of the genomic sequences flanking the coding region of *LjCDA1* implied that the activity of its bilateral retrotransposons may be responsible for the genetic exclusion between *LjCDA1* and *LjCDA1L1s*, the CNVs, and the diverse genome insertion of *LjCDA1* among individuals.

The evolution of the AID/APOBEC family is closely related to their functions. As early as 2007, lamprey *CDA1* and *CDA2* genes were found to be related to VLR rearrangement (39). Recently, direct evidence that CDA2 is responsible for the assembly of VLRB has been achieved by generating the CDA2 knockout larva in *L. planeri* (24). Here, we found that the expression of LjCDA2 is highly correlated with VLRB, suggesting that LjCDA2 should also be involved in the assembly of VLRB in *L. japonicum.* Although *CDA2* is a single copy in the lamprey genome, splicing forms can be generated by alternative splicing. Moreover, these splicing forms are mainly localized in the cytosol when they are overexpressed in 293T cells. This finding is consistent with AID which localized primarily in the cytoplasm when overexpressed in NIH3T3 cells at steady state (40). When in the cytoplasm, AID is stabilized by the HSP90 chaperone pathway and anchored with eEF1A1 to restrict its nuclear translocation (40–42). The import of AID is accomplished through an N-terminal nuclear localization signal (NLS) interacting with the adaptor importins before being imported to the nucleus to initiate the hypermutation and recombination of immunoglobulin genes. Once in the nucleus, the C-terminal NES signal of AID will lead its translocation from the nucleus to maintain a low level of AID in the nucleus to avoid genome instability (40). However, unlike mammalian AID, no NLS or NES signal sequences can be predicted from LjCDA2 by ProtParam. Thus, the study on the nuclear translocation of CDA2 accomplished by other proteins and the transcriptional regulations of CDA2 in VLRB-positive cells will help us to reveal the assembly mechanism of VLRB.

Besides CDA2, PmCDA1 was proposed to be involved in the assembly of VLRA/C. Since LjCDA1 does not exist in all *L. japonicum* individuals and its expression could not be detected in lymphocytes in individual Lj#5, we proposed that LjCDA1 may not participate in the assembly of VLRA/C in *L. japonicum*. A study on the AID/APOBEC family in *L. planeri* suggested that LpCDA1L1_4 may be functionally equal to vertebrate AID and PmCDA1, as all of them share a high net positive surface charge for efficient ssDNA substrate on/off binding (23). Although CDA1L1s are specifically distributed in gill slits, the thymus candidate in lamprey, here, we proposed that the LjCDA1L2 rather than LjCDA1L1_4s may play predominant roles in mediating VLRA/C assembly in lamprey due to the following considerations. First, the expression of LjCDA1L1 is rare in VLRA/C cells. Second, the deamination activity of LjCDA1L1_4a is extremely high, and it even can act on dsDNA. If such high enzyme activity in the nucleus is not well regulated, it may influence the genome stability of the lamprey. Third, LjCDA1L2 has a positive surface charge and equal enzymatic activity to HsAID. Moreover, LjCDA1L2 is highly conserved and restricted in the nucleus, and its expression is abundant in lamprey lymphocyte-like cells. Thus, further investigations on how distinct CDA1L2 isoforms are generated and whether they participate in the assembly of VLRA/C will help us to understand the assembly of VLRA/C.

If not participating in VLR assembly, the expansion of *LjCDA1L1s* may result from an efficient arms race between pathogens and host. First, the sequence alignment suggested that the zinc coordinating domain named as Z1 by LaRue et al. exits in mammalian A3A, A3B, A3G, and LjCDA1L1s (14). Second, like mammalian APOBEC3s which are organized in a tandem array between two vertebrate-conserved flanking genes, *CBX6* and *CBX7* (14), lamprey CDA1L1s have also undergone lineage-specific expansion in a tandem array. Third, like mammalian A3 whose copy numbers can vary greatly among species (14), sequence diversity and the CNVs of *LjCDA1L1* occur rapidly and dynamically among individuals and distinct lamprey species. Fourth, like APOBEC3s which are classical ISGs against viral infection (2), LjCDA1L1s can inhibit the infection of HSV-1. In the force of mammalian A3 proteins, the viruses have also evolved mechanisms to resist APOBEC3s’ mutations. For example, the nucleoprotein (NC) of human T-cell leukemia virus type 1 (HTLV-1) can inhibit the integration of A3G into HTLV-1 virus particles (43), while the Vif protein of HIV-1 can combine with HsA3G protein and other proteins like CBF-b and Cul5 to mediate the degradation of HsA3G in the proteasome (44, 45). Additionally, ribonucleotide reductase (RNR) large subunits of herpesviruses [Epstein–Barr virus (EBV), Kaposi’s sarcoma-associated herpesvirus (KSHV), and HSV-1] can also directly bind and localize with human APOBEC3B and APOBEC3A to protect viral DNA replication intermediates (46). Here, we found that although most of the initial mutations mediated by LjCDA1L1s in passage 1 were lost in the subsequent experimental evolution of HSV-1, some mutations were still accumulated under strong pressure from the APOBEC proteins. Thus, the APOBEC strategy through rapid expansion and sequence variation may be forced during the arms race between the host and viruses, similar to that found in lamprey, primates, and other distinct species.

In all, by reassessing the expanded AID/APOBEC in lamprey, we not only identified a cytidine deaminase with high activity on ssDNA and buDNA and, unexpectedly, on dsDNA, but also shed new light on the functional co-evolution of this family with VLR assembly and the host antiviral immune defense.

## Data Availability Statement

The datasets presented in this study can be found in online repositories. The names of the repository/repositories and accession number(s) can be found in the article/**Supplementary Material**.

## Ethics Statement

The animal study was reviewed and approved by the School of Life Sciences, Sun Yat-sen University.

## Author Contributions

SY and AX conceived and coordinated the project. SY, YC, and LL designed the experiments and analyzed the data. YC, LL, LD, and XT performed the experiments cooperatively. SC provided the discussion. SY drafted the manuscript. SY and AX edited and approved the submitted manuscript.

## Funding

This work was supported by the National Natural Science Foundation of China (31970852 and 31770943), Ministry of Science and Technology of the People’s Republic of China (2018YFD0900502), Guangdong Science and Technology Department (2017B030314021), and Innovation Group Project of Southern Marine Science and Engineering Guangdong Laboratory (Zhuhai) (311021006).

## Conflict of Interest

The authors declare that the research was conducted in the absence of any commercial or financial relationships that could be construed as a potential conflict of interest.

## Publisher’s Note

All claims expressed in this article are solely those of the authors and do not necessarily represent those of their affiliated organizations, or those of the publisher, the editors and the reviewers. Any product that may be evaluated in this article, or claim that may be made by its manufacturer, is not guaranteed or endorsed by the publisher.
