# Supplementary material for: New Insights Into the Lineage-Specific Expansion and Functional Diversification of Lamprey AID/APOBEC Family
Source: Front Immunol. 2022 Mar 11;13:822616. doi: 10.3389/fimmu.2022.822616 (PMC8962628; doi:10.3389/fimmu.2022.822616)
Supplement: Supplementary file 1 [file DataSheet_1.pdf]

Supplementary Material

This file includes:

Table S1. Primers used in this study.

Fig. S1. Neighbor-joining tree of lamprey and other AID/APOBEC deaminases.

Fig. S2. The alignment of the amino acid sequences of lamprey and human AID/APOBEC members.

Fig. S3. Genomic organization and alternative splicing of LjCDA2 and LjCDA1L2.

Fig. S4. Lamprey and human AID/APOBEC stimulated mutations of *rpoB* in *E. coli*.

Fig. S5. Cytidine deaminase activity of LjCDA1L1s on buDNA at different pH and temperatures *in vitro*.

Fig. S6. Cytidine deaminase activity of LjCDA1L1s on buDNA and dsDNA *in vitro*.

Fig. S7. The antiviral activity of LjCDA1L1 subgroup to HSV-1.

Table S1. Primers used in this study

Primer	Sequence
qLjGAPDH-F	5'-CAACCAACTGCCTGGCTCCT-3'
qLjGAPDH-R	5'-AGTCTTCTGCGTTGCCGTGTA-3'
qVLRB-F1	5'-TCGCTCCCCAACACTCTCA-3'
qVLRB-R1	5'-TCCACTTGATCCACATGATGGT-3'
qVLRA-F1	5'-CAAGGTCGTCAGAACAGTGACC-3'
qVLRA-R1	5'-GAACTCAGCCTGTGGCACTCTCA ACACG-3'
qVLRC-F1	5'-GCCGTCAAAGATGTTAATAC-3'
qVLRC-R1	5'-GAA AACATGCCACTTTATGAAATG-3'
qLjCDA1-F1	5'-CAATGGTGCTGGAACAACT-3'
qLjCDA1-R1	5'-TCTTAGTGGTGTGGAGTCTT-3'
qLjCDA2-F1	5'-GCCTACGGTCTGTTCCTA-3'
qLjCDA2-R1	5'-ACCTTGATAGTTGTGTTTCCTC-3'
qLjCDA1L1-F1	5'-CGGCG ACGAGAACGT GCGAG-3'
qLjCDA1L1-R1	5'-TCATGTAAAC AGGTGCAAGG-3'
qLjCDA1L2-F1	5'-CGGTCT TCCTCCACAAGAAG-3'
qLjCDA1L2-R1	5'-GA TCCTCAAGCG GCAGG-3'
US12-F	5'-CTCGTAGTAGACCCRAATCTCCACATT-3'
US12-R	5'-GCCGACGTACGCGATGAGATYAAT-3'
LjCDA2-FP	5'-ATGGAGCTCCGGGAGGTGGT-3'
LjCDA2-RP	5'-TCATGGTATATCATCTTCATGGCTCTGAG-3'

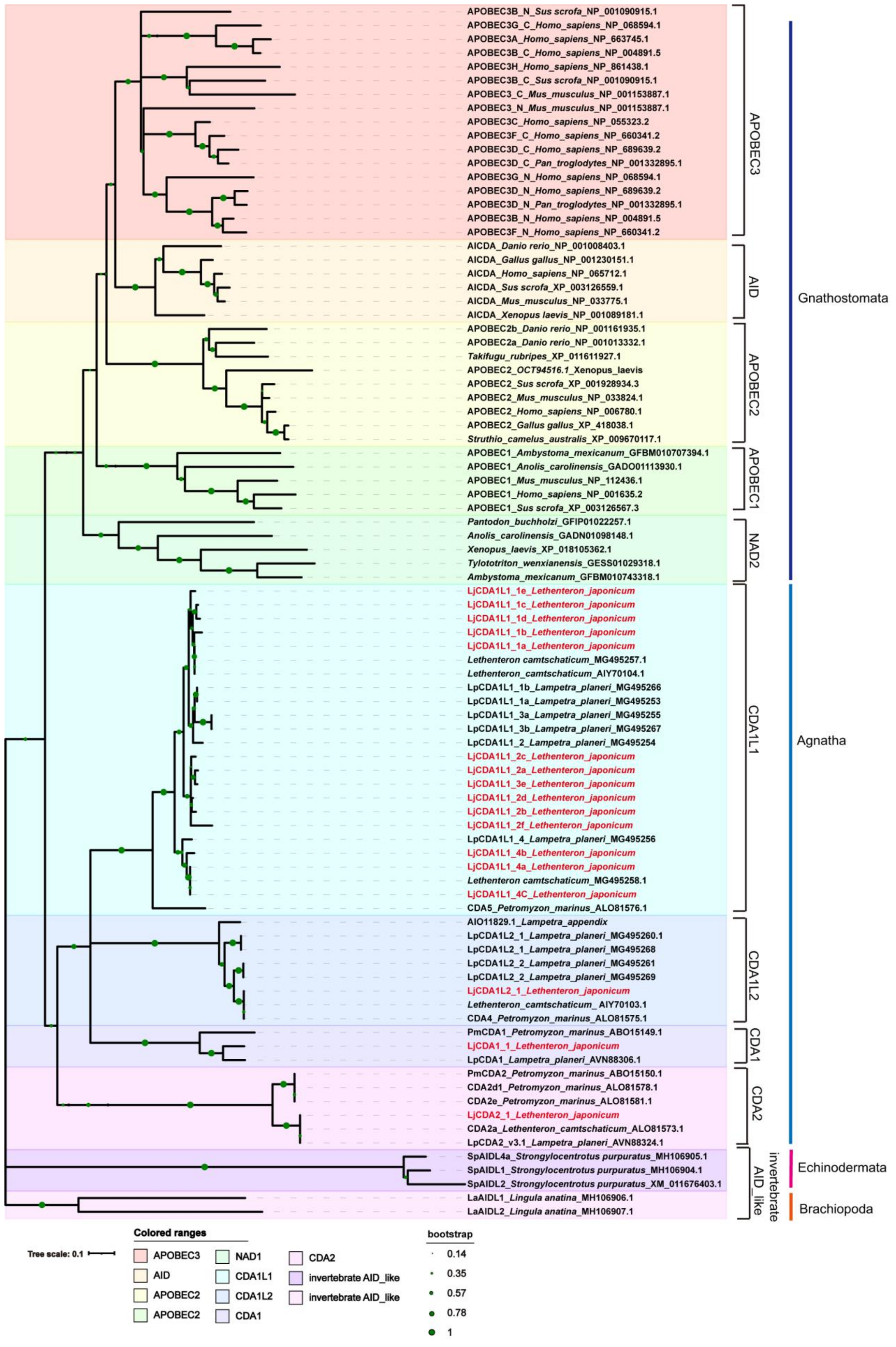

Fig.S1.Neighbor-joining tree of lamprey and the other AID/APOBEC deaminases.

The amino acid sequences of the core region of the cytidine deaminases (CDAs) were

aligned using CLUSTALW. Alignments were manually adjusted based on secondary structure predictions. A neighbor joining tree was calculated using MEGA version 5.05 with 1000 bootstrap replications. The number at each branch point indicates the bootstrap values for each branch point. The accession number of the cloned LjCDA1_1 is MT240960. The accession numbers of the cloned LjCDA1L1s are MT240947 to MT240951 and OM218945 to OM218953.

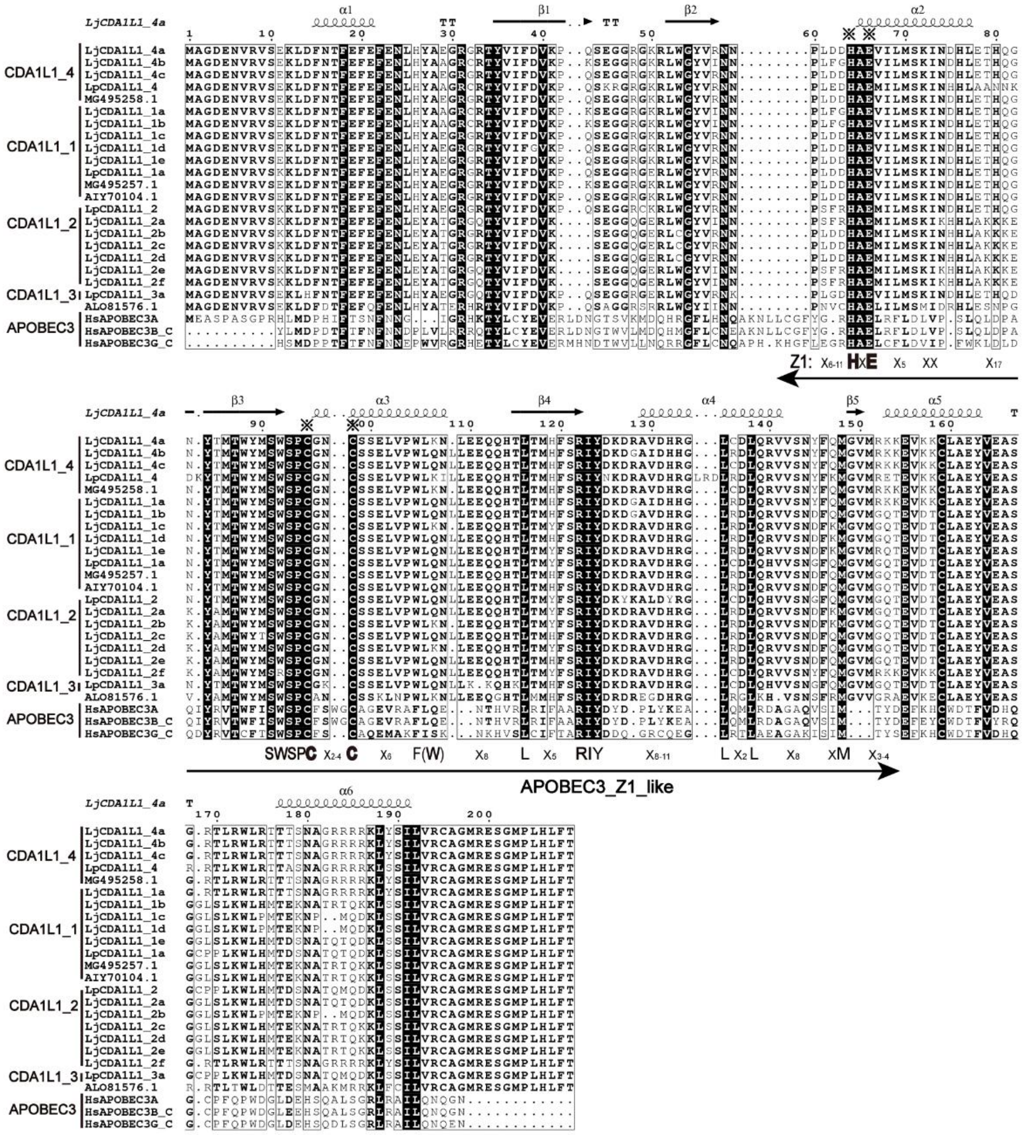

Fig. S2. The alignment of the amino acid sequences of LjCDA1L1s and human APOBEC3 members. ※ indicates the conserved amino acid of the activity center of CDA (cytidine deaminase); The conserved amino acids of Z1 domain are indicated. Hs: *Homo sapiens*; Lj: *Lethenteron japonicum*；Lp: *Lampetra planeri.*

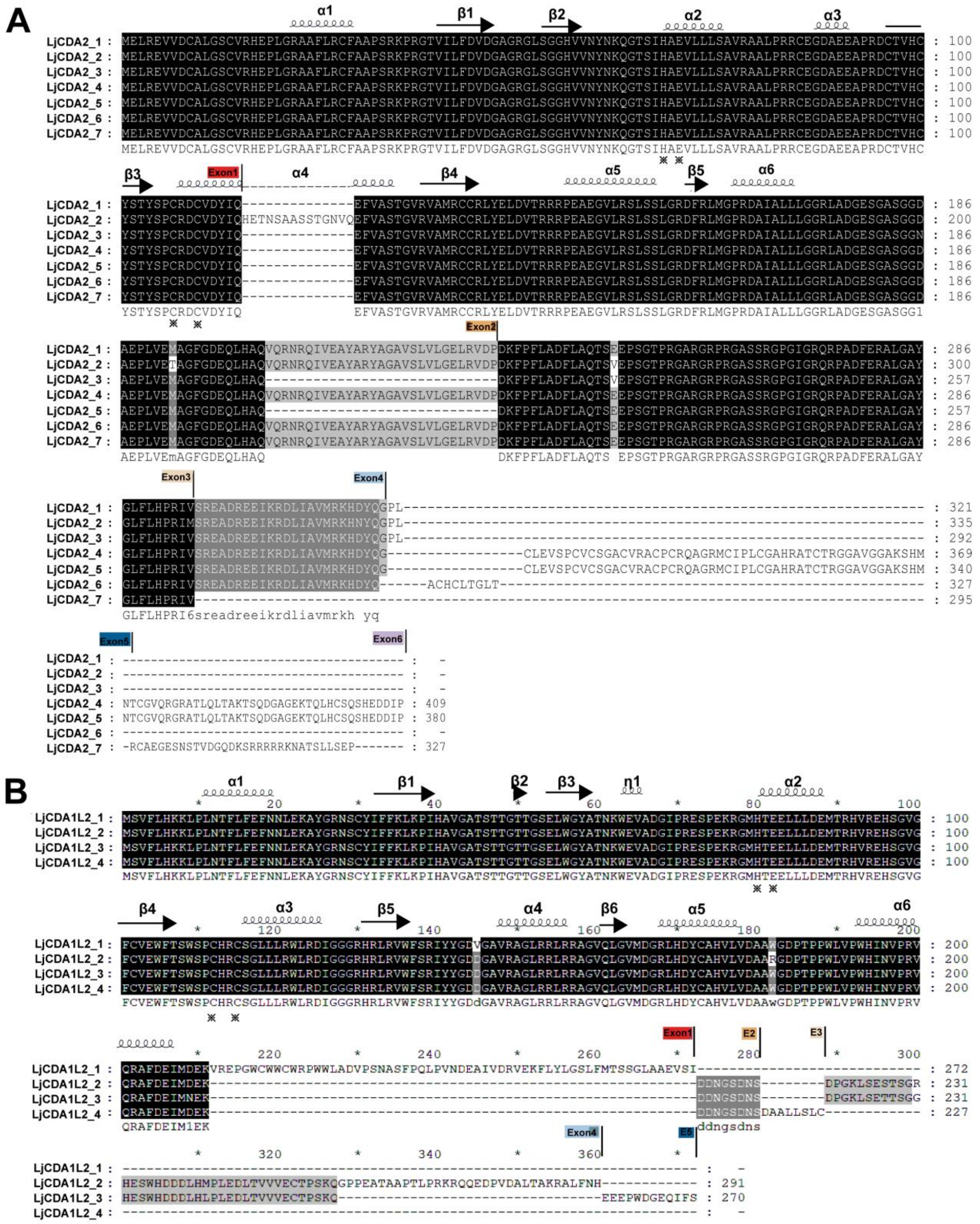

Fig. S3. Genomic organization and alternative splicing of LjCDA2 and LjCDA1L2. (A) Amino acids alignment of the spliced and unspliced LjCDA2 gene products from *L. japonicum.* The accession numbers of the cloned LjCDA2 and its spliced isoforms are from MT240956 to MT240959 and OM218954 to OM218956.**(B)** Amino acids alignment of the spliced and unspliced LjCDA1L2 gene products from *L.*

japonicum. The accession numbers of the cloned LjCDA1L2 and its spliced isoforms are from MT240952 to MT240955.

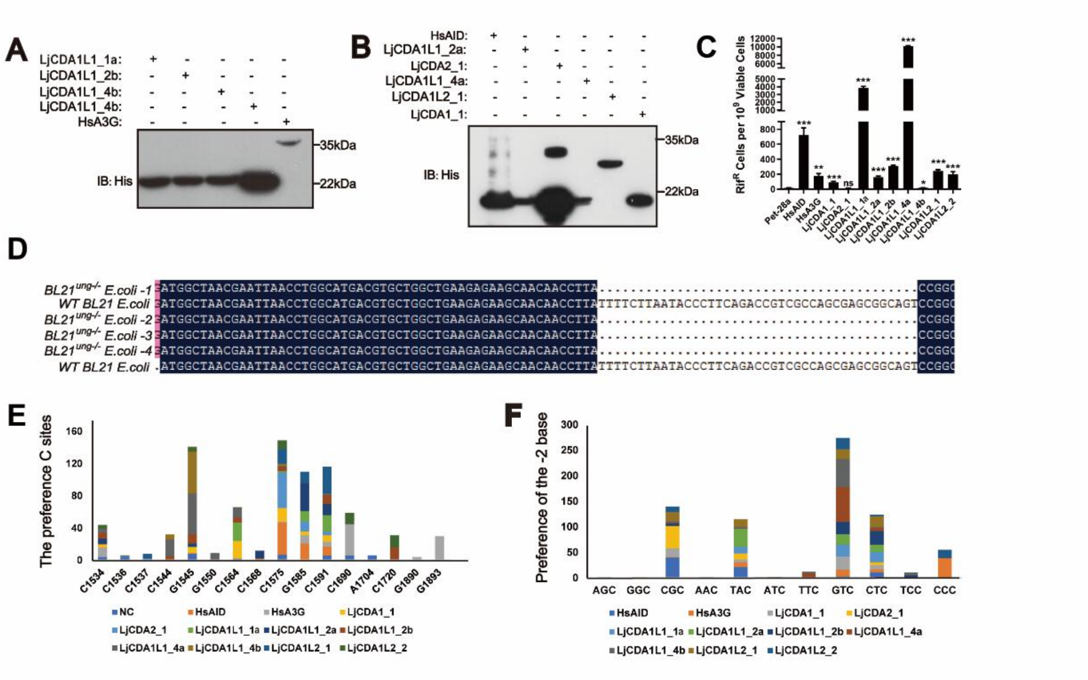

Fig. S4. Lamprey and human AID/APOBEC stimulated mutations of *rpoB* in *E. coli.* **(A, B)** Western blots to detect the expression of LjAID/APOBEC proteins in *E.coli* BL21. **(C)** Mutagenic activities of the LjAID/APOBEC proteins in *E.coli* BL21. Activity was measured as the number of rifampicin-resistant (Rif^R) colonies per 10^9 viable cells (number of kanamycin-resistant colonies). Deaminase activities were evaluated against the vector control using an unpaired *Student's t* test with Welch's correction. $^{***}P < 0.0001$, $^{**}P < 0.001$. **(D)** The nucleotide sequences of *ung* gene edited by cas9. **(E)** The higher mutation sites of *rpoB* gene edited by LjAID/APOBEC in BL21$^{ung-/-}$. X-axis represents the mutation base sites of *rpoB* gene. For example, C1534 represents the 1534th cytidylic acid of *rpoB* gene. Y-axis represents the number of mutations mediated by LjAID/APOBEC at distinct mutated sites. **(F)** The statistical analysis of the base preference of LjAID/APOBEC family proteins at the -2 position upstream of cytidine deamination site (0). Y-axis represents the number of mutations mediated by LjAID/APOBEC at distinct mutated sites.

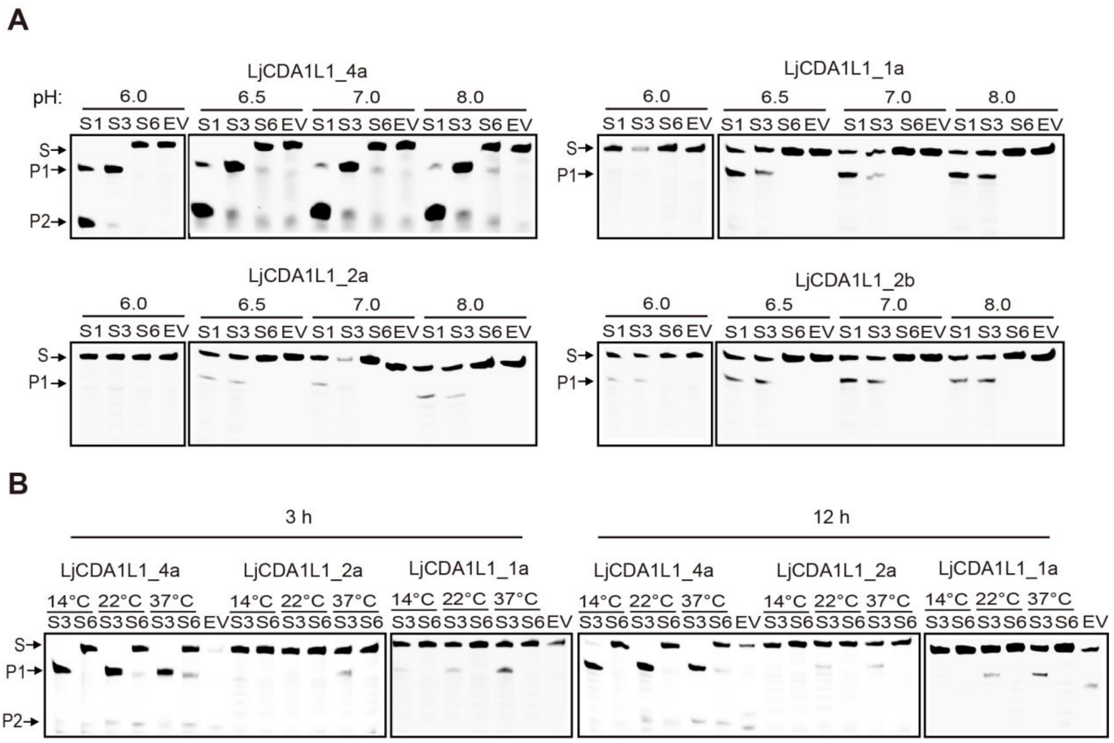

Fig. S5. Cytidine deaminase activity of LjCDA1L1s on buDNA at different pH and temperatures *in vitro*. (A) Cytidine deaminase activity of LjCDA1L1s on buDNA at different pH *in vitro*. Whole cell lysates of LjAID/APOBEC expressing 293T cells were used to test the enzyme activity at 37°C, pH ranging from 6.0 to 8.0. **(B)** Cytidine deaminase activity of LjCDA1L1s on buDNA at different temperatures *in vitro*. Whole cell lysates of LjAID/APOBEC expressing 293T cells were used to test the enzyme activity at 14°C, 22°C and 37°C for 3h or 12h. S1, S3 and S6 represent the buDNA substrates showed in Fig.4B. S, substrate; P, product; EV, empty vector. All results were representative of three independent biological replicate.

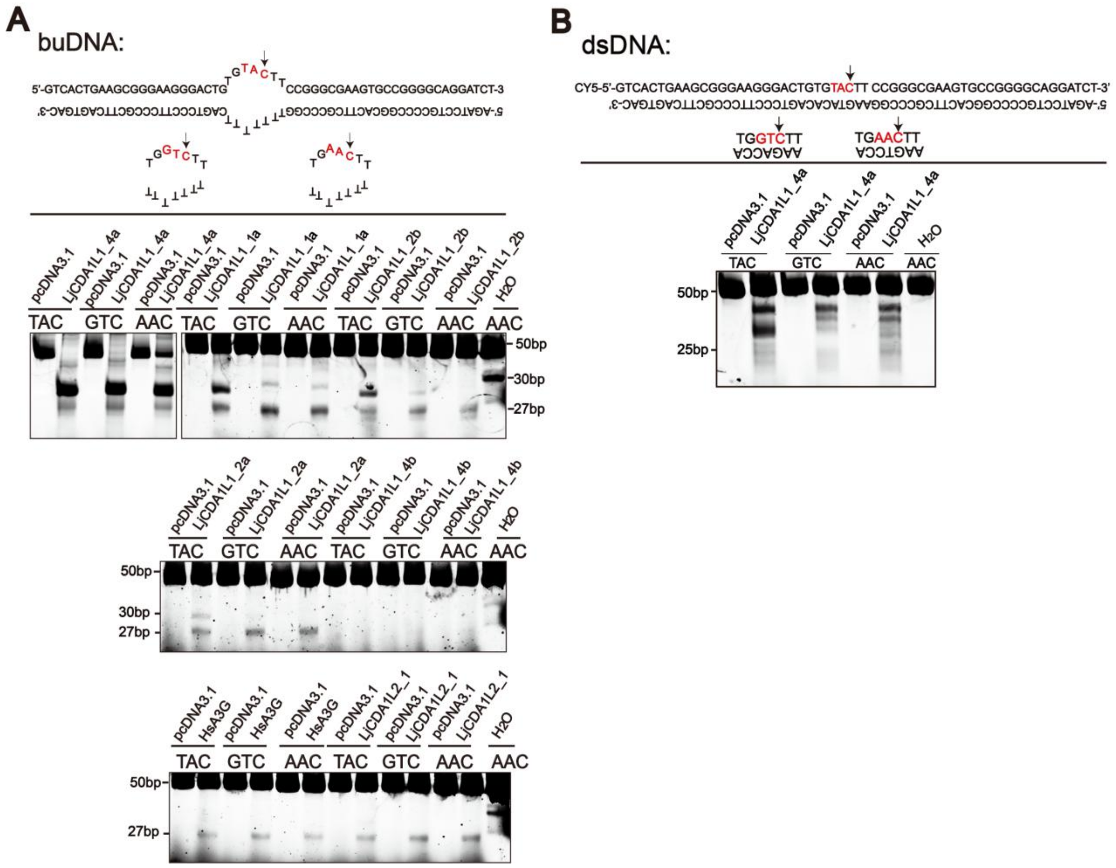

Fig. S6. Cytidine deaminase activity of LjCDA1L1s on buDNA and dsDNA *in vitro*. (A) Cytidine deaminase activity of LjAID/APOBEC on buDNA *in vitro*. Upper sequences are the used buDNA substrates. Arrows represent the target C. Whole cell lysates of LjAID/APOBEC expressing 293T cells were used to test the enzyme activity. **(B)** Cytidine deaminase activity of LjCDA1L1s on dsDNA. Upper sequences are the used dsDNA substrates. Arrows represent the target C. Whole cell lysates of LjAID/APOBEC expressing 293T cells were used to test the enzyme activity.

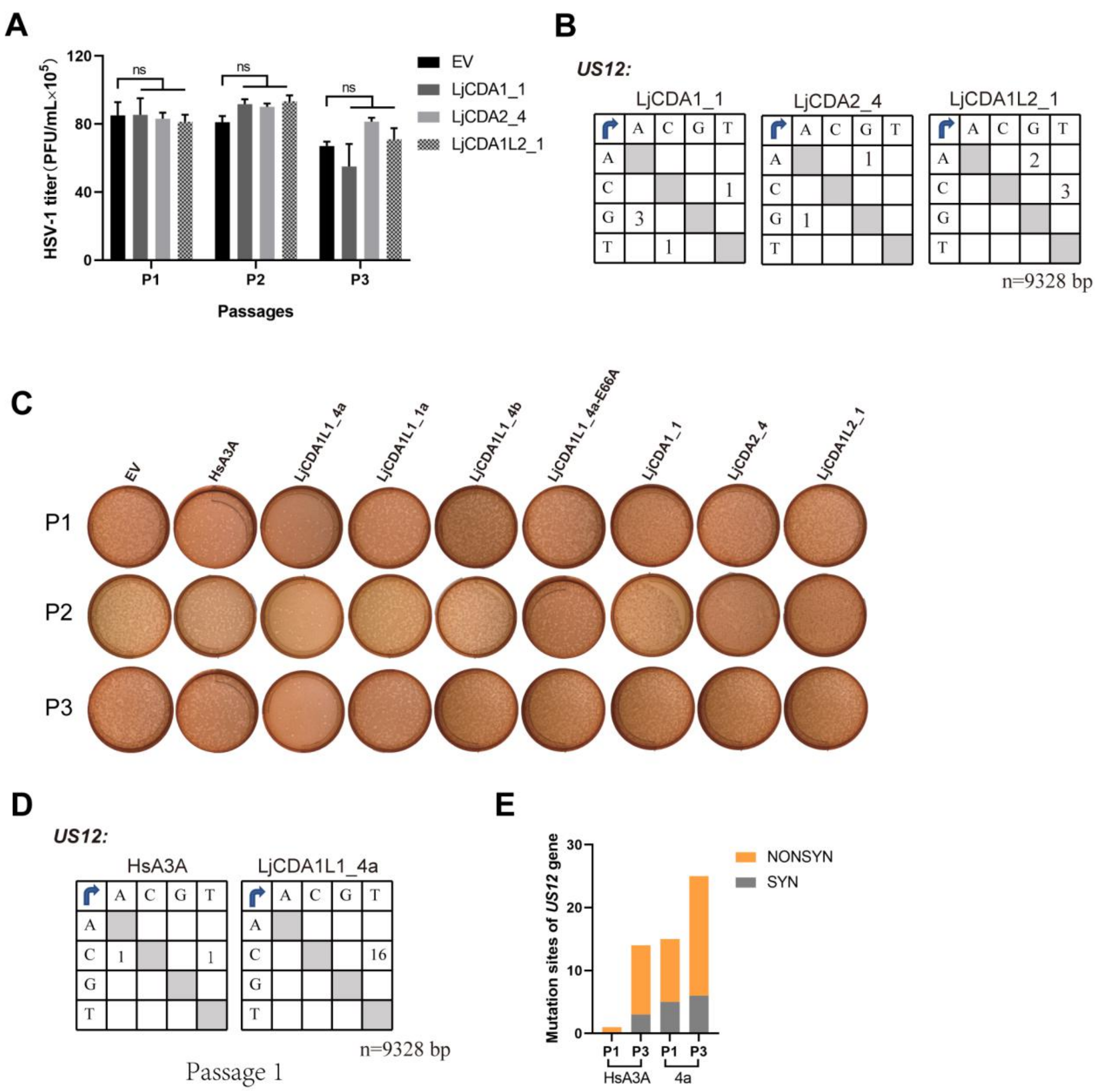

Fig. S7. The antiviral activity of LjCDA1L1 subgroup to HSV-1. (A) The PFUs of HSV-1 harvested from the supernatants of LjCDA1_1, LjCDA2_4 and LjCDA1L2_1 proteins-expressing 293T cells at 48 h after infection by HSV-1 at 0.1 MOI from three passages. Error bars denote ± SD. **(B)** Mutation matrices for *US12* gene of HSV-1 extracted from 293T cells expressing LjCDA1_1, LjCDA2_4 and LjCDA1L2_1 proteins from the third passages. **(C)** Plaques staining of HSV-1 harvested from the supernatants of LjAID/APOBEC and HsA3A proteins-expressing 293T cells at 48 h after infection by HSV-1 at 0.1 MOI from three passages. **(D)** Mutation matrices for *US12* gene of HSV-1 extracted from 293T cells expressing HsA3A and

LjCDA1L1_4a proteins from the first passages. n=9328bp. **(E)** The statistical analysis of synonymous and non-synonymous mutation sites for *US12* gene of HSV-1 extracted from 293T cells expressing HsA3A and LjCDA1L1_4a proteins from the 1st and 3rd passages.
